# Rhizosphere Ventilation Effects on Root Development and Bacterial Diversity of Peanut in Compacted Soil

**DOI:** 10.3390/plants13060790

**Published:** 2024-03-11

**Authors:** Haiyan Liang, Liyu Yang, Xinhua He, Qi Wu, Dianxu Chen, Miao Liu, Pu Shen

**Affiliations:** 1 Shandong Peanut Research Institute/Key Laboratory of Peanut Biology, Genetics & Breeding, Ministry of Agriculture and Rural Affairs, Shandong Academy of Agricultural Sciences, Qingdao 266100, China; 15094073735@163.com (H.L.); liyuyang@whu.edu.cn (L.Y.); qi_wu@126.com (Q.W.); chenpeanut@126.com (D.C.); liumiao123123@163.com (M.L.); 2 School of Biological Sciences, University of Western Australia, Perth 6009, Australia; xinhua.he@uwa.edu.au; 3 Department of Land, Air and Water Resources, University of California at Davis, Davis, CA 90616, USA

**Keywords:** nitrogen-fixing bacterial communities, peanut, root morphology, root enzyme activity, soil ventilation

## Abstract

Soil compaction is one of the crucial factors that restrains the root respiration, energy metabolism and growth of peanut (*Arachis hypogaea* L.) due to hypoxia, which can be alleviated by ventilation. We therefore carried out a pot experiment with three treatments: no ventilation control (CK), (2) ventilation volumes at 1.2 (T1), and 1.5 (T2) times of the standard ventilation volume (2.02 L/pot). Compared to no-ventilation in compacted soil, ventilation T1 significantly increased total root length, root surface area, root volume and tips at the peanut anthesis stage (62 days after sowing), while T2 showed a negative impact on the above-mentioned root morphological characteristics. At the podding stage (S2, 95 days after sowing), both ventilation treatments improved root morphology, especially under T1. Compared to CK, both ventilation T1 and T2 decreased the activities of enzymes involving the anaerobic respiration, including root lactate dehydrogenase, pyruvate decarboxylase and alcohol dehydrogenase. The activities of antioxidant enzymes of root superoxide dismutase, peroxidase and catalase also decreased at S1, while superoxide dismutase and peroxidase significantly increased under T1 at S2. The ventilation of compacted soil changed soil nitrogen-fixing bacterial communities, with highest bacterial alpha diversity indices under T1. The Pearson correlation analyses indicated a positive relationship between the relative abundance of *Bradyrhizobiaceae* and root activity, and between unclassified_family of *Rhizobiales* and the root surface area, while *Enterobacteriaceae* had a negative impact on the root nodule number. The Pearson correlation test showed that the root surface, tips and activity positively correlated with root superoxide dismutase and peroxidase activities. These results demonstrate that soil ventilation could enhance plant root growth, the diversity and function of soil nitrogen-fixing bacterial communities. The generated results from this present study could serve as important evidence in alleviating soil hypoxia caused by compaction.

## 1. Introduction

Peanuts (*Arachis hypogaea* L.), important nitrogen (N)-fixing crops, play an important role in soil fertility and fertilizer input to soil [1]. As an underground seed crop, peanut roots are sensitive to soil hard pan or compaction layer, seriously affecting the growth of peanuts. In recent years, soil compaction due to intensive agriculture and mechanization has become an increasingly serious problem [2,3,4]. Compacted soil usually has poor air and higher mechanical impedance than uncompacted soils [5,6], affecting the root systems and penetration capacity of crops including maize [7], tomato and wheat [8,9]. The high CO_2_ and low O_2_ concentrations in compacted soil restrict the aerobic respiration of roots and thus restrict crop growth and development [10,11].

Studies have indeed demonstrated that soil compaction has harmful effects on plant root morphology and growth, water and nutrient absorption, carbohydrate contents and enzyme activity [7,12]. The reason is that both rhizosphere soil permeability and root penetration capacity are poor under compaction; thus, the ability of root nutrient absorption and plant growth are negatively affected [13]. Studies have shown that soil ventilation or injecting additional oxygen directly into rhizosphere has been applied to improve root length and root vitality [14,15] while removing excess subsurface gas (including CO_2_) [16,17]. For example, by using a compressor to inject air into rhizosphere or the subsurface drip irrigation system is an effective way to improve water absorption by potato [16], metabolism in tomato [18], and yields of wheat, potato, soybean, cotton, tomato and muskmelon [14,16,19,20,21,22]. Nakano [23] and Xiao et al. [13] found that tomato root activity and absorptive capacity were improved by rhizosphere ventilation because of the improvement in rhizosphere gas diffusion. Ventilation also alleviated soil hypoxia and increased pepper and pomegranate yields [24,25]. Different from other nonlegume, peanut root has the ability to form a symbiotic relationship with soil bacteria collectively called rhizobia, and thus has biological nitrogen fixation ability [26]. And as a unique underground seed legume, peanut roots are more sensitive to deleterious environmental conditions (such as low soil O_2_) than other crops, which affects the root growth and yield [27]. However, the effects of ventilation on peanut rhizosphere are unknown. There has been little empirical work conducted on analyzing the effect of soil ventilation on peanut root morphology, activity and enzymatic activities under compacted soil.

Microorganisms inhabiting the rhizosphere play an important role in plant nutrition and growth [28,29,30], as well as responses to stress [31]. Soil bacteria need to consume O_2_ for performing normal physiological activity and respiration, while low O_2_ concentration in compacted soil limits such activities. Indeed, it is expected that ventilation can improve the aerobic condition of rhizosphere soil, with an increase in microbial biomass in tomato and sugarcane rhizosphere soil [21,32]. The abundance of *Acidobacteria* was increased and *Gammaproteobacteria* decreased with soil rhizosphere aeration [22]. Qian et al. [33,34] reported that ventilation increased soil bacterial diversity and nutrient transformation under mulching-induced hypoxic conditions. What is more, rhizobia in the soil are closer to peanut roots, which can reside in the root organ to form nodules [26,27]. These rhizobia are capable of biological nitrogen fixation from atmospheric gas (N_2_) to enhance N absorption and utilization. Torabian et al. [27] reported that increased soil compaction makes inappropriate soil condition for rhizobia proliferation and nodulation. Soil management affected the relative presence of specific microorganisms and activity, with reports of increases in the number of rhizobial cells [35,36]. However, the influence of ventilation on soil bacterial community composition and soil nutrients in compacted peanut soil is not known. A better understanding of the linkage between soil ventilation and bacterial communities is vital for a sustainable peanut ecosystem.

To fill these knowledge gaps, we hypothesized that soil ventilation by burying silicone tubes can affect rhizosphere bacterial community composition, improve root morphology, root enzyme activity and nutrient adsorption in compacted soil. Therefore, the following goals were set: (1) determine the responses of soil bacterial communities, particularly the nitrogen-fixing bacterial community, to soil ventilation; and (2) evaluate the effects of ventilation on root properties, including growth, root enzyme activity and nutrient absorption. To provide evidence for testing the abovementioned hypotheses, plant performance, root physiology and 16S rRNA gene sequencing were applied to assess the effects and relationships of artificial rhizosphere soil ventilation on peanut root morphology, root enzyme and the bacteria dynamics of community composition.

## 2. Materials and Methods

### 2.1. Experimental Site

The present pot experiment was conducted inside a greenhouse located at the Laixi Experimental Station (36°48′47″ N, 120°30′17″ E), Shandong Peanut Research Institute, China, from July to October of 2022. This region consists of a temperate monsoon climate, with a mean annual temperature of 11.7 °C and a mean annual precipitation of 635.8 mm, respectively. The soil is classified as alfisol (based on the USA soil taxonomy) that developed from lime rock, which was collected (0–20 cm) under the peanut agricultural field and had the following properties: pH 5.9; total N 0.9 g/kg; available P 96.7 mg/kg; available K 79 mg/kg; organic C 9.7 g/kg.

### 2.2. Experimental Design

**Figure 1:**
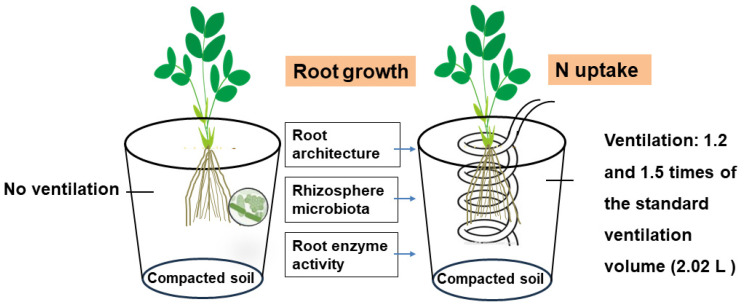
A conceptual diagram showing the effect of rhizosphere ventilation on root growth and soil microbial communities.

The <2 mm air-dried soil was repacked into a plastic container of 22 cm height and 18 cm inner diameter to give a depth of 20 cm. Before placing the soil, the intake tube was placed in the containers in a spiral pattern to achieve a uniform soil distribution. The intake tube had holes with a diameter of 2 mm for every 5 cm. A layer of silk wool covered the holes to prevent the entrance of soil into the venthole. Then, the container was packed with the air-dried soil layer-by-layer and the tubes protruded 20 cm from the soil surface to be connected to an air compressor. The entire soil layer of the container had a bulk density of 1.6 g/cm^3^ to produce the compacted conditions. There were three ventilation treatments with three replicates, CK, T1 and T2, representing no ventilation, and 1.2 and 1.5 times of the standard ventilation volume (2.02 L/pot), respectively (Figure 1). The flow rate for each treatment was 3 L/min, and the ventilation frequency was once every 3 days. The airway was closed after ventilation to prevent gas leakage. The standard ventilation volume was calculated following the equation outlined by Li et al. (2016b) [21] as follows:

$$V=\frac{1}{1000}SL(1-\frac{\rho_{b}}{\rho_{s}})$$

where *V* is the volume (L) of air injected in each treatment, *S* is the cross-sectional (254.34 cm^2^) of the pot, *L* is the depth of the tube in the soil (20 cm), *ρ_b_* is the soil bulk density (1.60 g cm^−3^), and *ρ_s_* is the soil particle density (2.65 g cm^−3^). Accordingly, the calculated standard ventilation volume was 2.02 L.

The *A. hypogaea* ‘Huayu 22’, which was used in this study, is one of the dominant varieties in Shandong province, east China. The seeds of peanut were extensively rinsed 3 times with distilled water, and then germinated in glass petri dishes under dark at 28 °C for three days. Germinated seeds were planted into soil and two seedings per pot were selected after emergence. Artificial ventilation for all of the treatments was started 30 days after germination.

### 2.3. Sample Collection

The soil, root and aboveground tissues were respectively collected during the anthesis (S1, 62 days after emergence) and podding periods (S2, 95 days after emergence), as these two periods are critical in peanut growth and the most active period of nitrogen-fixing bacteria in soil. For plant samples, each of the three replicate samples for each treatment were composited from the two individual plants per pot with a similar size and then separated as roots, leaves and stems. After removing the aboveground tissues, using a 30 mm soil auger, five soil cores from a 0–20 cm depth and at a 10 cm distance from the main stem of the harvested plants were randomly collected as one composite bulk soil sample. Then, the samples were immediately transported to the laboratory, sieved (<2 mm) after the removal of debris and divided into two subsamples. The first subsample was stored at −80 °C for soil microbiological high-throughput sequence analysis; the second was air-dried and stored at room temperature for soil nutrient content analyses. After the soil sampling, a mixture of roots and soil was firstly soaked on a 100-mesh steel sieve, and then gently washed to separate the roots from the soil. The collected roots were dried with absorbent paper and analyzed for root morphology, nodule number and activity. The plant samples were transported to the laboratory and then separated into stems, leaves and pods (at podding stage), which were dried at 105 °C for 30 min and then at 75 °C to achieve content weight. After the relative biomasses were measured, plant samples that were grounded into powder were stored at room temperature until total N analysis.

### 2.4. Root Measurements

The obtained root samples were placed in transparent trays filled with water to a depth of 1–2 mm. Then, transparent trays were scanned with an Espon scanner (modified Optical Scanner STD 4800, Epson, Matsumoto, Japan) to obtain a grey-scale image. This image was analyzed with WinRHIZO Regular 2009 software (Regent instruments Inc., Quebec, QC, Canada) to obtain the total root length (cm), total surface area (cm^2^), total root volume (cm^3^) and root tips.

After root morphology analysis, a part of the fresh root samples was used to assess root activities. Total superoxide dismutase (SOD) activity was measured using the nitroblue tetrazolium (NBT) method [37], Guaiacol peroxidase (POD) activity was assayed as described by Omran [38]. Catalase (CAT) activity was determined by the consumption of H_2_O_2_ (extinction coefficient 39.4 mM^−1^ cm^−1^) at 240 nm for 3 min, as described by Guan [37]. Root activity (DHA) was measured using the triphenyltetrazolium chloride method (TTC method) [39], and the content of malondialdehyde (MDA) was analyzed using the thiobarbituric acid (TBA) method [39]. Ethanol dehydrogenase (ADH), pyruvate dehydrogenase (PDH) and lactate dehydrogenase (LDH) were determined as reported by Li [40].

### 2.5. Soil Nutrient Measurements

Soil chemical properties of available N (AN), available P (AP) and available K (AK) were analyzed as per the previously described protocols [41]. Briefly, AN was measured using the NaOH-hydrolysisdiffusion method, AP was determined by molybdenum blue spectrophotometry and AK was measured by using the flame photometry method.

### 2.6. DNA Extraction, PCR Amplification, and High-Throughput Sequencing

Total genomic DNA was extracted from soil samples using the TGuide S96 Magnetic Soil/Stool DNA Kit (Tiangen Biotech Co., Ltd., Beijing, China), according to the manufacturer’s instructions. The quality and quantity of the extracted DNA were examined using electrophoresis on a 1.8% agarose gel, and DNA concentration and purity were determined with an NanoDrop 2000 UV-Vis spectrophotometer (Thermo Scientific, Wilmington, CA, USA). The hypervariable region V3–V4 of the bacterial 16S rRNA gene was amplified with primer pairs nifHF: 5′-TGCGAYCCSAARGCBGACTC-3′ and nifHR: 5′-ATSGCCATCATYTCRCCGGA-3′ [42]. Both the forward and reverse 16S primers were tailed with sample-specific Illumina index sequences to allow for deep sequencing. The PCR was performed in a total reaction volume of 10 μL: DNA template 50 ng, forward primer (10 μM) 0.3 μL, reverse primer (10 μM) 0.3 μL, KOD FX Neo Buffer 5 μL, dNTP (2 mM each) 2 μL, KOD FX Neo 0.2 μL, and finally ddH_2_O up to 20 μL. Initial denaturation at 95 °C for 5 min was followed by 30 cycles of denaturation at 95 °C for 30 s, annealing at 55 °C for 30 s, an extension at 72 °C for 40 s, and a final step at 72 °C for 7 min. The amplicon library was paired-end-sequenced (2 × 250) on an Illumina novaseq6000 (Beijing Biomarker Technologies Co., Ltd., Beijing, China). A negative control was run with sterilized distilled water, and the amplification resulted in single peaks with efficiencies of 92% and R^2^ values of 0.995. The 16S rRNA sequencing files were deposited in the Sequence Read Archives (SRA) of NCBI with the accession numbers of BioSample SAMN36085943-SAMN36085948, BioPriject PRJNA989381.

### 2.7. Sequence Data Analysis

MiSeq sequencing generated paired sequence data, and the bioinformatics analysis was performed by the aid of the BMKCloud (http://www.biocloud.net/, (accessed on 1 June 2023)). According to the quality of single nucleotides, raw data were primarily filtered by Trimmomatic [43]. The identification and removal of primer sequences were processed by Cutadapt [44]. After obtaining demultiplexed reads trimmed of the primer and index sequences, paired-end sequences were processed by using DATA2 (version 1.8.0) [45], which inferred unique bacterial taxa from amplicon sequence variants (ASVs). There were 79,865 clean reads per sample. A total of 439,575 high-quality sequences were obtained and classified as amplicon sequence variants (ASVs) by the amplification and sequencing of 16S rRNA genes from all samples. The coverages of bacterial communities were greater than 99% in all treatments, representing a sufficient sequencing depth to describe the diversity of bacterial communities. The high-quality reads generated from the above steps were used in the following analysis. In this study, the amplicon sequence variants of ASVs were performed by the Naive Bayes classifier in QIIME2 using the SILVA database with a confidence threshold of 70%. The alpha diversity of soil bacterial communities was analyzed based on the Chao1, Shannon and Simpson diversity indexes, and it performed to identify the complexity of species diversity of each sample utilizing QIIME2 2020.6.0 software. The beta diversity calculations were analyzed by principal coordinate analysis (PCoA) to assess the diversity in the samples for species complexity. A one-way analysis of variance was used to compare the bacterial abundance and diversity.

### 2.8. Statistical Analysis

The root morphology, root enzyme activities and soil nutrient content data were expressed as the means ± standard (SD) of triplicates. And we used a one-way ANOVA followed by the Fisher’s least significant differences (*LSD*) test to determine statistical significance at *p* < 0.05. All statistical analyses were conducted using the SPSS version 23.0 (SPSS for Windows, IBM Corp., Chicago, IL, USA). All figures were constructed using the graphing software SigmaPlot 12.5 (software, Systat Inc., San Jose, CA, USA).

The alpha diversity of soil bacterial communities was assessed based on the Chao1, Shannon and Simpson diversity indexes. The Venn diagrams and redundance analysis (RDA) were all generated using the relative abundance, and each ASV was divided by the sequencing depth for each sample. The subsequence diversity analysis was performed based on these normalized data using the free online platform of BMKCloud (http://www.biocloud.net/, (accessed on 1 June 2023)). The Pearson correlation was conducted between the root morphology index and bacterial communities at the genus level.

## 3. Result

### 3.1. Composition of Soil Bacterial Communities in Peanut Soil

**Figure 2:**
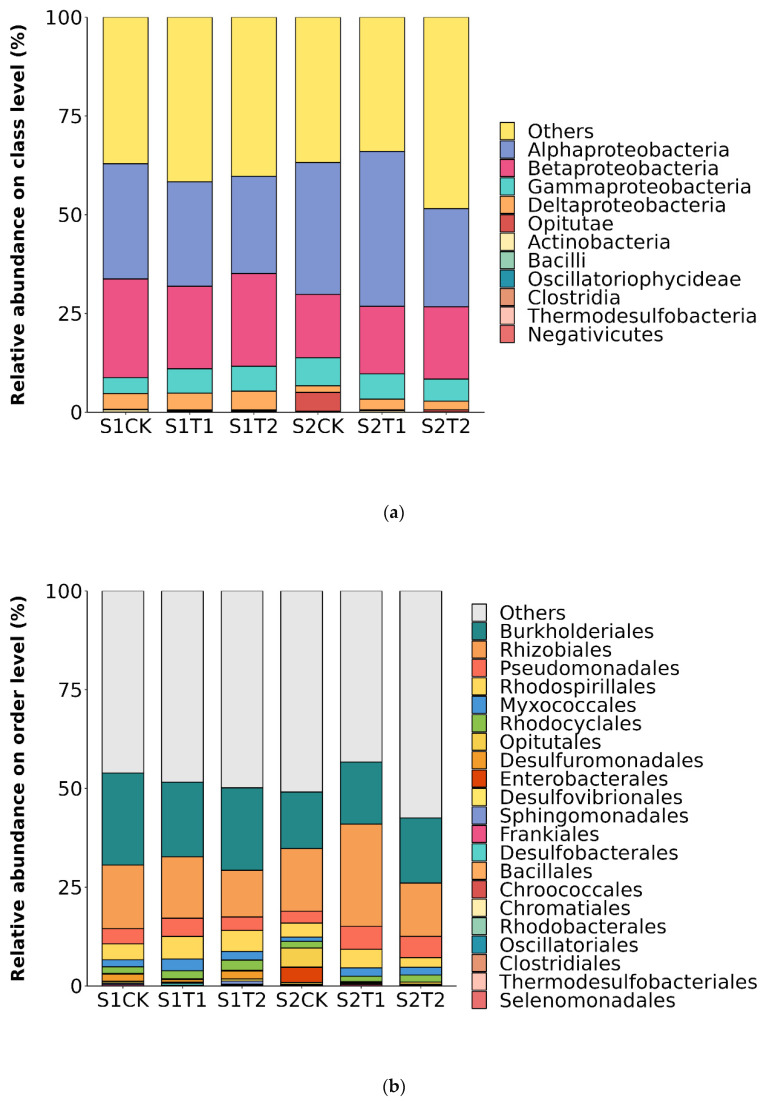
Soil bacterial community structure at the class (**a**) and order (**b**) levels under different ventilation treatments for the anthesis stage (S1) and podding stage (S2).

We analyzed the relative abundances of peanut soil bacteria in the three treatments at the class (a) and order (b) classification levels (Figure 2), at the anthesis stage (S1) and podding stage (S2). At the class level, the relative abundance of bacteria displayed a decreasing trend in the bacterial communities of T1 and T2 treatments at the S1 stage. *Alphaproteobacteria* (24.81–39.24%), *Betaproteobacteria* (16.01–24.98%), *Gammaproteobacteria* (4.02–7.14%) and *Deltaproteobacteria* (1.65–4.24%) were the dominant bacterial classes (Figure 2a). At the S1 stage, the two ventilation treatments decreased the relative abundance of bacterial communities compared to CK. On the other hand, at the S2 stage, the relative abundance of bacteria, particularly *Alphaproteobacteria*, *Betaproteobacteria* and *Deltaproteobacteria*, was higher under T1 than under CK and T2. The dominant bacterial orders were *Burkholderiales* (14.33–23.30%), *Rhizobiales* (11.83–25.91%), *Pseudomonadales* (2.99–5.81%) and *Rhodospirillales* (2.44–5.71%) (Figure 2b). At the S1 stage, the relative abundances of *Rhizobiales* and *Rhodospirillales* were higher in ventilation treatments than in the no-ventilation treatment, whereas at the S2 stage, the relative abundances of *Burkholderiales* and *Pseudomonadales* were higher under T1 and T2 treatments than under CK.

**Figure 3:**
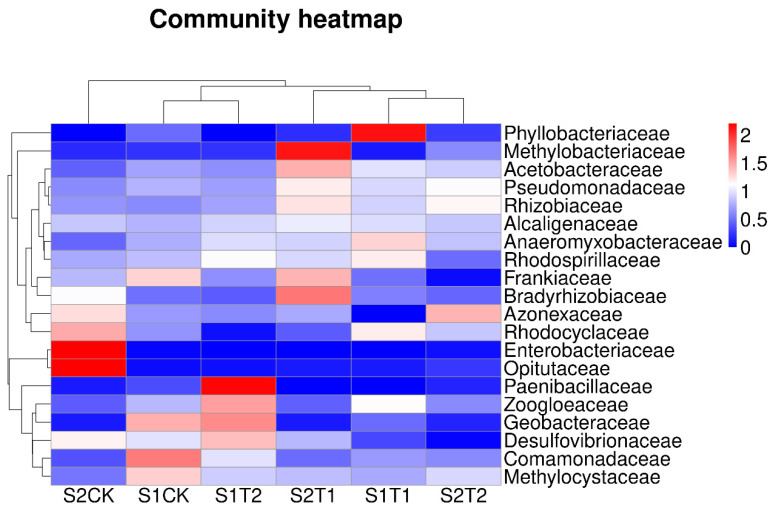
Variations in soil bacterial distribution of the top 20 abundance families between soil ventilation treatments based on the family level. S1, anthesis stage; and S2, podding stage.

The 20 most abundant bacterial species at the family level of each treatment in terms of relative abundance are shown in Figure 3. The hierarchical heat map illustrates that soil bacterial community compositions of T1 at the S1 stage were significantly different from those of T2, and also from those of CK treatment. At the S2 stage, soil bacterial community compositions of T1 and T2 were significantly different from those of the CK treatment. According to the PCoA analysis (Figure S1), there were significant differences in soil bacterial community compositions among the three treatments at the S2 stage.

### 3.2. Root Morphology, Biomass, Nodule Number and N Accumulation

**Figure 4:**
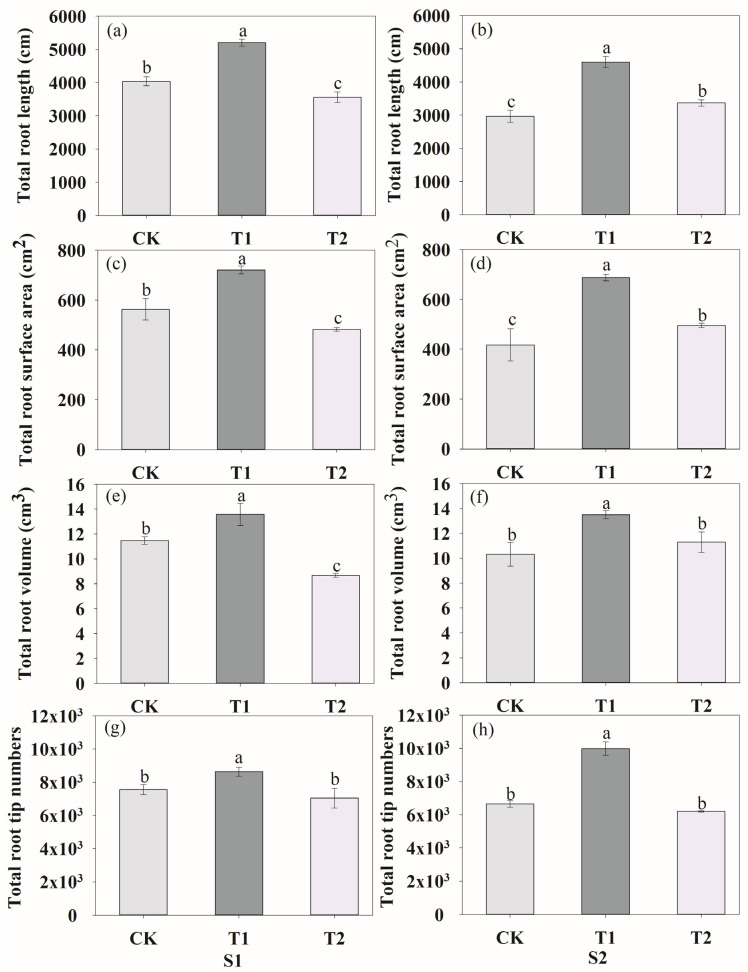
Effects of rhizosphere ventilation on the total root length (**a**,**b**), total root surface area (**c**,**d**), root volume (**e**,**f**) and root tip numbers (**g**,**h**) of peanut under different ventilation treatments at S1 and S2. S1, anthesis stage; and S2, podding stage. Different letters above the bars indicate a significant difference at *p* < 0.05 level.

**Figure 5:**
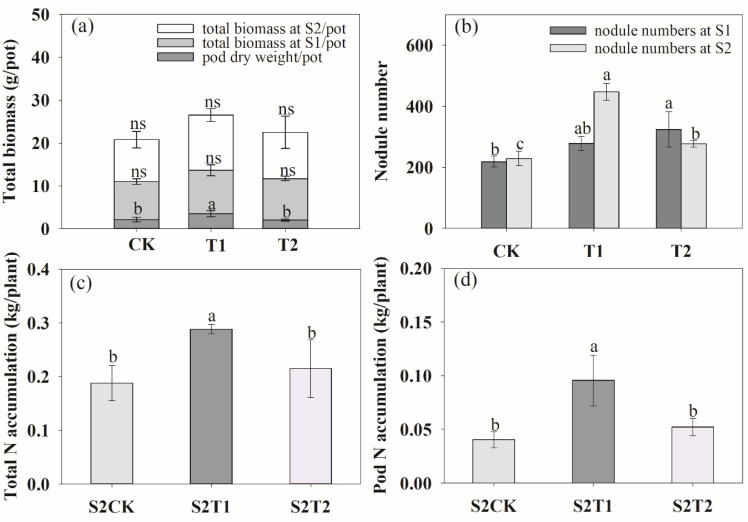
Effects of rhizosphere ventilation on total biomass (**a**), root nodule number (**b**), plant total N accumulation (**c**) and pod N accumulation (**d**) of peanut under different ventilation treatments. S1, anthesis stage; and S2, podding stage. Different letters above the bars indicate a significant difference at *p* < 0.05 level.

We analyzed the total root length, root surface area, root volume and root tips at the anthesis stage (S1) and podding stage (S2). At the S1 stage, ventilation in T1 treatment significantly increased the total root length (Figure 4a), root surface area (Figure 4c), root volume (Figure 4e) and tips (Figure 4g) by 28.79%, 28.05%, 18.46% and 14.14%, respectively. Inversely, compared to CK, T2 decreased root length, root surface area, root volume by 11.94%, 14.43% and 24.28%, respectively, with no significant impact on root tips. During the two growth stages, the root length, root surface area and root volume decreased when the ventilation volume increased. At the S2 stage, both ventilation treatments increased the root growth (Figure 4b,d,f,h), while T1 had the highest effect. Rhizosphere ventilation treatments improved the plant biomass and development of nodule number during the two growth stages (Figure 5). The pod dry weight per pot significantly increased under both ventilation treatments, especially under T1, compared to CK (Figure 5a). In comparison with CK, the nodule number of T1 and T2 increased by 27.33%, 48.40% and 95.63%, 21.14%, respectively, during the two growth stages (Figure 5b). The rhizosphere ventilation of peanut under soil compaction increasd root growth and thus nutrient uptake. Compared to the CK, the total N accumulation of plants under T1 and T2 increased by 53.55% and 14.28%, respectively (Figure S2).

### 3.3. Effect of Rhizosphere Ventilation on Enzyme Activities in Peanut

**Figure 6:**
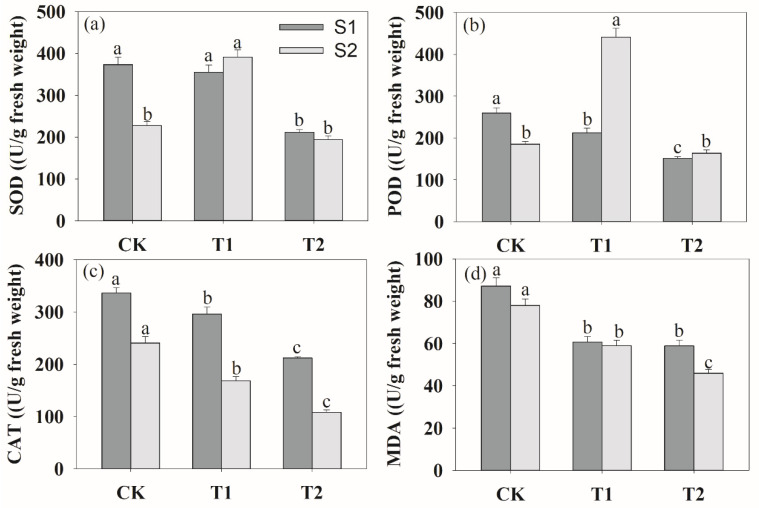
Effects of rhizosphere ventilation on the root protective enzymes of SOD (**a**), POD (**b**), CAT (**c**) and MDA content (**d**) under soil compacted conditions. SOD, superoxide dismutase; POD, peroxidase; CAT, catalase; MDA, malonaldehyde content. Different letters above the bars indicate a significant difference at *p* < 0.05 level.

The higher T2 rhizosphere ventilation decreased the activities of root superoxide dismutase (SOD), peroxidase (POD), catalase (CAT) and malonaldehyde content (MDA) at the S1 stage (Figure 6). At the S2 stage, the changes in CAT activity and MDA concentrations had a similar trend at the S1 stage under different ventilation. The activities of SOD and POD increased, and then decreased by increasing the ventilation volume.

**Figure 7:**
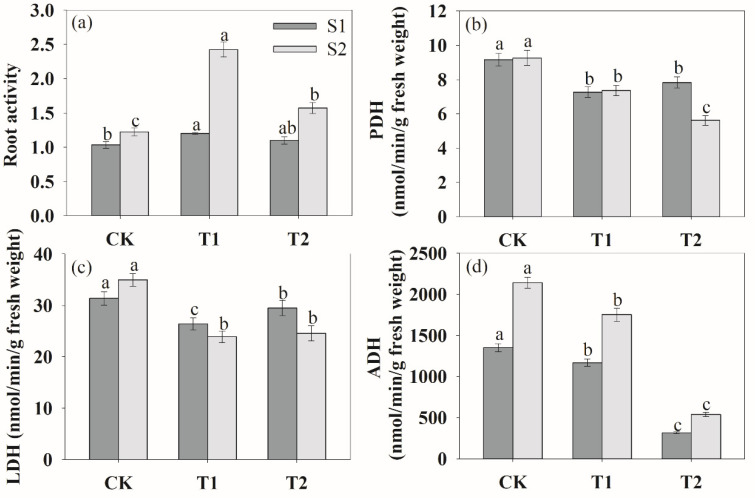
Effects of rhizosphere ventilation on root activity (**a**), PDH (**b**), LDH (**c**) and ADH (**d**) under soil compacted conditions. PHD, pyruvate decarboxylase; LDH, lactate dehydrogenase; ADH, alcohol dehydrogenase. Different letters above the bars indicate a significant difference at *p* < 0.05 level.

**Figure 8:**
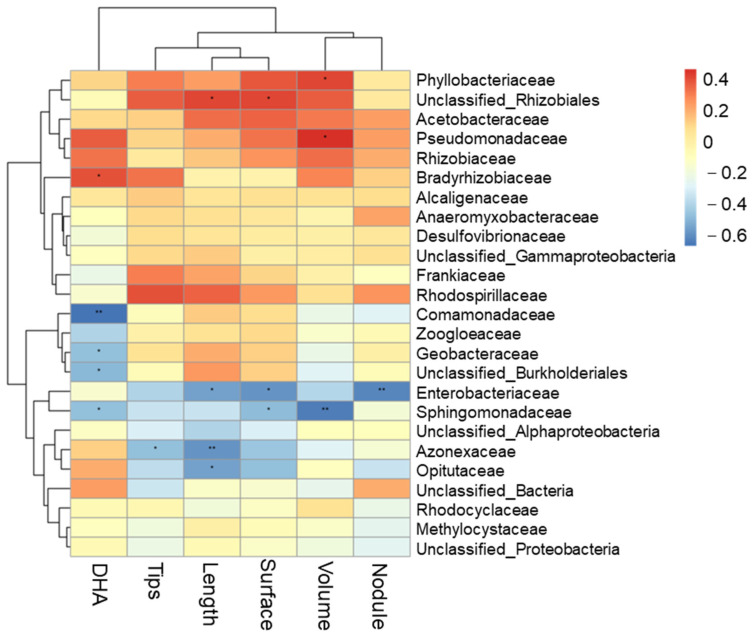
Pearson’s correlation coefficients of root morphology, DHA (root activity), nodule number and soil microbial communities at the genus level with a relative abundance > 1%. * or **, significances at the 5% or 1% levels.

We also analyzed root activity, lactate dehydrogenase (LDH), pyruvate decarboxylase (PDH) and alcohol dehydrogenase (ADH) under soil compaction (Figure 7). During the two periods of growth, rhizosphere ventilation significantly increased the root activity compared to the CK (Figure 7a). And the order of the effects of different ventilation volumes on root activity was T1 > T2 > CK. In addition, at the S2 stage, as the root began to accelerate senescence, rhizosphere ventilation treatments could significantly enhance root activity compared to the S1 stage. Figure 8 shows that the activities of PDH, LDH and ADH were the highest under CK treatment. And these enzymes decreased markedly among ventilation treatments during the two growth stages (Figure 7b–d).

### 3.4. Relationships between the Root Morphology, Root Enzymes and Soil Bacterial Communities of Peanut

The Pearson correlation analyses suggested that root morphology showed positive correlation with the relative abundance of the dominant genus (Figure 8). The total root length was significantly positively correlated with soil unclassified_*Rhizobiales* relative abundance, and was significantly negatively correlated with *Enterobacteriaceae*, *Azonexaceae* and *Opitutaceae*. Contrastingly, root activity (DHA) was significantly positively correlated with the relative abundance of *Bradyrhizobiaceae*, and was significantly negatively correlated with *Comamonadaceae*, *Geobacteraceae*, unclassified_*Burkholderiales* and *Sphingomonadaceae*, while the nodule number was significantly negatively correlated with the relative abundance of *Enterobacteriaceae*. The root surface area was significantly positively correlated with unclassified_*Rhizobiales* relative abundance. In addition, as shown in Table 1, the Pearson correlation analyses suggested that the root surface and root tip indicated a significant and positive correlation with the enzyme of SOD; and root tip, root activity had a significant and positive correlation with the enzyme of POD. The root nodule was significantly positively correlated with the Simpson and Shannon index. Root nitrogenase indicated a positive correlation with ventilation volume and was significantly and negatively correlated with the enzyme of ADH. Furthermore, we found that the enzyme activities of PDH, ADH and MDA were significantly negatively correlated with ventilation volume.

## 4. Discussion

In the present study, we investigated the nitrogen-fixing bacteria community in compacted soil under different ventilation treatments. Our results showed that rhizosphere ventilation led to significant changes in soil bacterial community diversity. Studies also showed that the diversity of bacterial communities in soil revealed different patterns under different rhizosphere ventilation [21,22,34]. We found that *Burkholderiales* and *Rhizobiales* were the dominant bacteria (Figure 2a), and some species of them can be symbiotically associated with leguminous plants [46]. Furthermore, the dominant family in the bacterial communities showed great differences between the two growing stages of S1 and S2 (Figure 3), indicating that there were significant differences in the community structure among the three treatments at the S2 stage (Figure S1), we thus found that the diversity of nitrogen-fixing bacteria was sensitive to soil ventilation. Rhizosphere ventilation significantly affected the composition of the bacterial community, especially as the proportion and amount of shared ASVs of all three ventilation treatments (2.75–4.13%; 85–144 ASVs) were less than the unique ASVs harbored by each ventilation (89.76–91.83%; 2843–3130 ASVs), which was contrary to the patterns found by Sun et al. [47] and Li et al. [22] in the entire bacterial community. This finding demonstrates that the nitrogen-fixing bacterial community may display different mechanisms for responding to changes in soil ventilation.

In addition, we found that *Bradyrhizobium*, *Azospirillum* and *Azotobacter* were the most abundant genera in T1 treatment than in CK and T2 treatment at the S1 stage, and at the S2 stage, *Bradyrhizobium* and *Azospirillum* were the most abundant indicator species, not considering the unclassified bacteria (Figure S3), which indicated that appropriate O_2_ contents were required for bacterial growth. On the contrary, the relative abundance of various taxa above decreased in T2 with excessive ventilation. Steenhoudt and Vanderleyden [48] already reported that *Azospirillum* was the best characterized genus of plant growth promoting rhizobacteria, and other studies have reported that *Bradyrhizobium* was frequently found to be symbiotic with peanuts [49,50], which plays an important role in N fixation in acid soil [42,51]. Thus, our results indicated that soil ventilation has the potential advantage in maintaining the structure and community of soil beneficial flora, especially under compacted soil. Our results were consistent with the findings of Li et al. [22], who reported that artificial soil ventilation could increase bacterial diversity. Alongside that, during the two growing stages, the response of bacteria to soil ventilation volume was different, and a proper ventilation volume was beneficial to increase the bacterial abundance that was related to root morphology and root activity (Figure 8).

Overall, in line with our hypothesis, proper soil ventilation promoted root growth, increased nutrient availability and altered the bacterial community structure. Soil ventilation regulation could be adopted as a strategy to promote sustainable peanut production under compacted soil with appropriate ventilation circumstances. In addition, we are aware of the limitation of this study. Due to the sample size and pot experiment, further studies are required to test the response of microbial (including bacteria and fungi) community structure and function to ventilation under long-term compacted soil.

### 4.1. Effects of Rhizosphere Ventilation on Root Morphology and Root Enzyme Activities

Root growth is directly related to plant growth and development. In our study, at the S1 stage, we obtained similar results for peanut, which was consistent with Li et al. [14] and Niu et al. [15]. An appropriate volume of rhizosphere ventilation under compacted soil significantly increased the root length, root surface area, volume and tips at two growth stages of peanut. However, when the ventilation of volume continues to increase, the root length, root surface area and volume decreased significantly, but there was no effect on root tips at S1. During S2, the root length and surface area significantly increased with the increasing ventilation volume and there was no impact on root volume and tips (Figure 4). The reason for these results is possibly due to the demand for oxygen of peanut root being different at different growth stages.

Under soil compaction stress, rhizosphere hypoxia inhibited plant root physiological functions [52], crop root tricarboxylic acid circulation was significantly hindered and anaerobic respiration metabolism was enhanced [53]. In the present study, anaerobic respiratory enzymes of LDH, PDH, LDH and ADH were the highest in CK treatment, whereas they obviously decreased after ventilation (Figure 7). And the changes in the antioxidant enzyme system (SOD, POD, CAT) and MDA content were similar to those of anaerobic respiratory enzymes (Figure 6). This indicated that ventilation can significantly alleviate the damage of anaerobic respiratory metabolites on roots and improve root growth. In other words, the results demonstrated that the negative impact of deficit on the measured responses can be offset by soil ventilation, especially with a proper ventilation volume. Moreover, we found that soil ventilation can effectively increase root activity. Ventilation volume was negatively correlated with MAD content, PDH ADH activities (Table 1). This was consistent with the results of Bhattarai et al. [19] and Niu et al. [15] on rhizosphere ventilation of potted tomato and rice. Overall, this study provides evidence that ventilation can effectively alleviate hypoxia in the peanut root zone. We assumed that rhizosphere ventilation with a reasonable volume would supply more O_2_ for plant root aerobic respiration under compacted soil and that this would improve plant root growth.

### 4.2. Effects of Rhizosphere Ventilation on Nodule Development and Plant Growth

The O_2_ relations with root nodules are extremely important in maintaining normal nodule development and functions [54]. And nitrogen fixation in mature nodules requires a large amount of ATP and high rate of respiratory O_2_ consumption by nodules. However, in compacted soil, hypoxia can limit the invasion of rhizobium flora to the roots, and thereby affect the root nodule development and nitrogen fixation [27]. In our study, the results demonstrated that rhizosphere ventilation significantly increased the root nodule numbers compared to the CK, which increased with the increasing ventilation volume (Figure 5). This may be due to the improvement in rhizosphere ventilation, which increased the diversity and abundance of rhizobium flora, and ultimately promoted nodule development. This can also be explained by the significant correlation between the Simpson and Shannon index and nodule numbers (Table 1). These results further indicated that rhizosphere ventilation can significantly relieve the inhibition of nodule development under compaction stress. But quite remarkably, the more rhizosphere ventilation is not a better strategy, because the plant rhizosphere has both aerobic azotobacter and amphitropic anaerobic bacteria, and a diffusion barrier in legume root nodules restricts O_2_ flux into the nodule and thereby protects nitrogenase from inactivation by O_2_ [55,56]. This study was consistent with the results from previous studies [56,57] considering root morphology and nodule development under soil ventilation.

Soil ventilation is a key factor that affects soil nutrient transformation and plant growth [58]. Our study revealed that the ventilation effectively enhanced the N accumulation of total plants. In other words, soil ventilation had no effects on N accumulation in the leaves, stems and roots, but this was significantly different in pod N accumulation (Figure S2). In comparison with CK, T1 treatment significantly increased the pod N accumulation, and this was closely related to the higher pod weight under T1 treatment (Figure 5). This results mainly because ventilation can significantly improve soil nutrient conditions by making nutrients readily available to plants and beneficial soil microbial [34,59]. Rhizosphere ventilation creates a good environment for the root system, and improves root growth and absorption. The increasing soil O_2_ content can promote soil nitrification. Additionally, the high amount of AN, AP and AK under T1 treatment resulted in a higher pod weight than that under CK treatment (Figure S1). In addition, soil ventilation has a significant impact on soil enzyme activity [60,61]. This may be another reason for improving plant N accumulation and pod yield, which may be attributed to the enhancement of soil enzyme activity, and further change in metabolic activity of microbials, improving the nutrient uptake of plants [21,62].

Soil ventilation offset the negative effect of hypoxia conditions on peanut root growth, soil bacterial communities and diversity, and therefore, could potentially improve plant growth and pod weight compared to that of no ventilation under compacted soil. Further field-scale experiments to validate these indicative relationships between soil ventilation, plant growth, nutrition and soil microorganism are suggested, taking into consideration the soil pH, temperature and tillage management as so on, as the field and greenhouse environments are more complex.

## 5. Conclusions

According to our results, soil ventilation promoted root length, surface area, volume, tips and root activity. The nodule numbers also increased obviously, especially under T1 treatment. Therefore, soil ventilation also increased the pod dry weight with appropriate ventilation volume, which induced soil nutrient transformation and plant absorption. In comparison with the control, when soil ventilation was applied, the enzyme activities of root pyruvate decarboxylase, lactate dehydrogenase, alcohol dehydrogenase and malonaldehyde content all decreased, meanwhile, the activity of root antioxidase enzymes of superoxide dismutase, peroxidase and catalase also decreased at the anthesis stage. On the other hand, the activity of superoxide dismutase and peroxidase at the podding stage were the highest under T1 because of root senescence. Soil ventilation had significant influences on soil nitrogen fixing bacterial abundance and diversity in peanut root zone. Soil bacterial alpha diversity indexes under T1 were the highest compared with the control. The Pearson correlation test results revealed that the root surface area, root tips and activity were positively correlated with root superoxide dismutase and peroxidase activities. These results suggest that soil ventilation can ameliorate hypoxic conditions under soil compaction, alter the diversity, composition, structure of soil nitrogen-fixing bacterial communities, and thus improve plant nutrient absorption and transportation, as well as root growth and pod weight.

## Figures and Tables

**Table 1:** Pearson’s correlation test for root morphology, root enzymes and soil bacterial α-diversity index.

	Length	Surface	Volume	Tips	Nodule	Activity	SOD	POD	CAT	MDA	PDH	LDH	ADH	ACE	Chao1	Simpson	Shannon	Ventilation Volume
Length	1																	
Surface	0.9831 **	1																
Volume	0.8034	0.8729 *	1															
Tips	0.8203 *	0.8704 *	0.7485	1														
nodule	0.3884	0.4915	0.3452	0.7164	1													
Activity	0.1741	0.3426	0.4691	0.5869	0.8112	1												
SOD	0.8082	0.8253 *	0.7661	0.8537 *	0.3046	0.3415	1											
POD	0.5582	0.6698	0.6937	0.8933 *	0.7234	0.8297 *	0.7936	1										
CAT	0.3479	0.2192	0.0962	0.1527	−0.5064	−0.5742	0.5192	−0.0385	1									
MDA	−0.1055	−0.1688	−0.1107	−0.0166	−0.5167	−0.3229	0.3663	0.0501	0.8044	1								
PDH	−0.1798	−0.2535	−0.2656	0.0013	−0.3857	−0.3266	0.2449	0.0157	0.7354	0.9412 **	1							
LDH	−0.5506	−0.6418	−0.5764	−0.4794	−0.6781	−0.5912	−0.2417	−0.4496	0.5469	0.7886	0.8618 *	1						
ADH	0.0479	0.103	0.3904	0.3676	−0.0095	0.2909	0.4463	0.4911	0.2988	0.5849	0.5873	0.3837	1					
ACE	0.2609	0.4234	0.6917	0.3915	0.4858	0.8099	0.2925	0.6126	−0.5607	−0.4455	−0.6002	−0.7271	0.1865	1				
Chao1	0.2609	0.4234	0.6919	0.3915	0.4855	0.8098	0.2926	0.6126	−0.5605	−0.4453	−0.6	−0.7269	0.1867	1.0000 **	1			
Simpson	0.3091	0.3498	0.0458	0.4482	0.8676 *	0.4708	0.0043	0.3286	−0.5269	−0.7074	−0.5329	−0.675	−0.4556	0.1852	0.1848	1		
Shannon	0.3635	0.5113	0.5724	0.5991	0.8668 *	0.8960 *	0.2533	0.694	−0.6804	−0.66	−0.648	−0.8423 *	0.0411	0.8377 *	0.8375 *	0.6624	1	
Ventilation volume	−0.0219	−0.0062	−0.2162	−0.1537	0.412	0.1068	−0.4843	−0.2441	−0.69	−0.9040 *	−0.8199 *	−0.6392	−0.8451*	0.1313	0.131	0.75	0.4084	1

Note: significant level: * = *p* < 0.05, ** = *p* < 0.01. Length, total root length; surface, total root surface area; volume, total root volume; tips, total root tips; nodule, root nodule number; activity, root activity; SOD, superoxide dismutase; POD, peroxidase; CAT, catalase; MDA, malonaldehyde content; PHD, pyruvate decarboxylase; LDH, lactate dehydrogenase; ADH, alcohol dehydrogenase; Shannon diversity index, Chao index and ACE index.

## Data Availability

Data will be made availability on request. The datasets generated during the current study have been submitted to the National Center for Biotechnology Information (NCBI) Sequence Read Archine (SRA) database under accession number PRJNA989381 (NCBI reviewer link: https://www.ncbi.nlm.nih.gov/sra/PRJNA989381, (accessed on 1 June 2023)).

## Supplementary Materials

The following supporting information can be downloaded at: https://www.mdpi.com/article/10.3390/plants13060790/s1, Figure S1: Principal coordinate analysis (PCoA) plot derived from unweighted Unifrac distances metrics of nitrogen-fixing bacterial communities (a). Venn diagrams display the shared and specific ASVs among the ventilation treatments in the growth stage of (b) antheses, (c) podding; Figure S2: Effects of rhizosphere ventilation on N accumulation of peanut organs under soil compacted conditions; Figure S3: Circos diagram represents the bacterial composition of top genus level under different ventilation conditions; Table S1: Effects of different ventilation treatments on soil physicochemical properties.
