# Supplementary material for: Rhizosphere Ventilation Effects on Root Development and Bacterial Diversity of Peanut in Compacted Soil
Source: Plants (Basel). 2024 Mar 11;13(6):790. doi: 10.3390/plants13060790 (PMC10975058; doi:10.3390/plants13060790)
Supplement: Supplementary file 1 [file plants-13-00790-s001.zip › plants-2838370-supplementary.pdf]

plants

MDPI

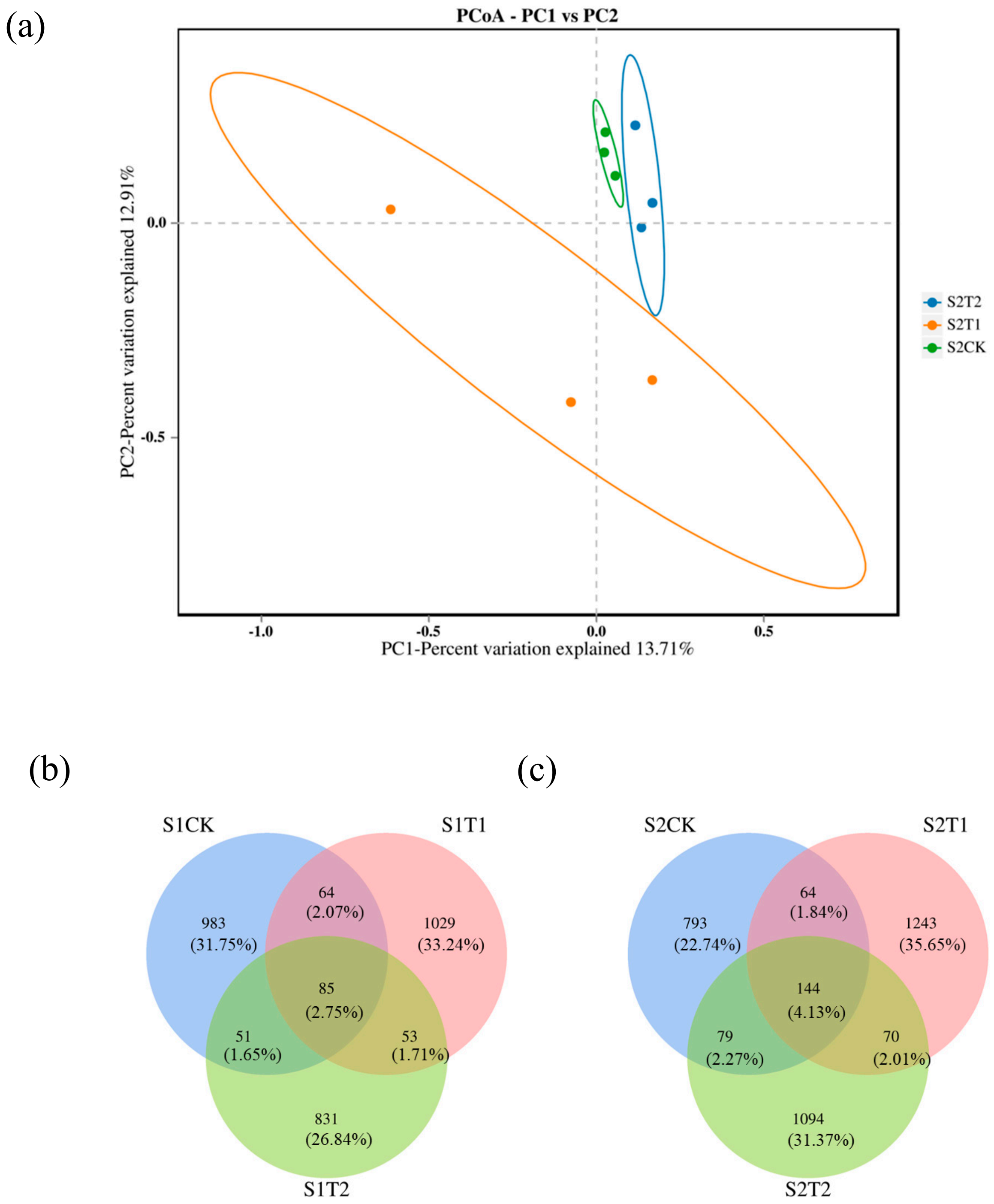

Figure S1 Principal coordinate analysis (PCoA) plot derived from unweighted Unifrac distances metrics of nitrogen-fixing bacterial communities (a). Venn diagrams display the shared and specific ASVs among the ventilation treatments in the growth stage of (b) antheses, (c) podding. CK, no ventilation; T1 the ventilation volume is 1.2 times as the standard; T2, the ventilation volume is 1.5 times as the standard; S1, anthesis stage; S2, podding stage.

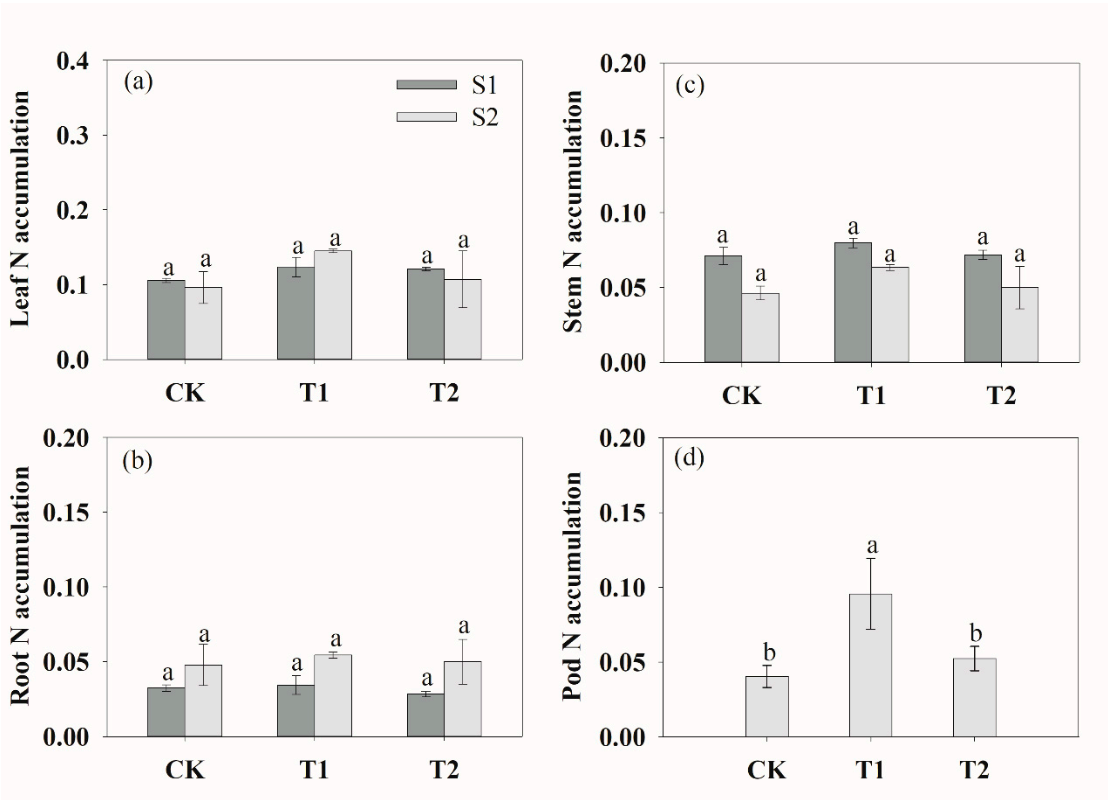

Figure S2 Effects of rhizosphere ventilation on N accumulation of peanut organs under soil compacted conditions. Leaf N (a), stem N (b), root N (c), pod N (d). CK, no ventilation; T1 the ventilation volume is 1.2 time as the standard; T2, the ventilation volume is 1.5 times as the standard; S1, anthesis stage and S2, podding stage. Different letters above the bars indicate a significant difference at p<0.05 level.

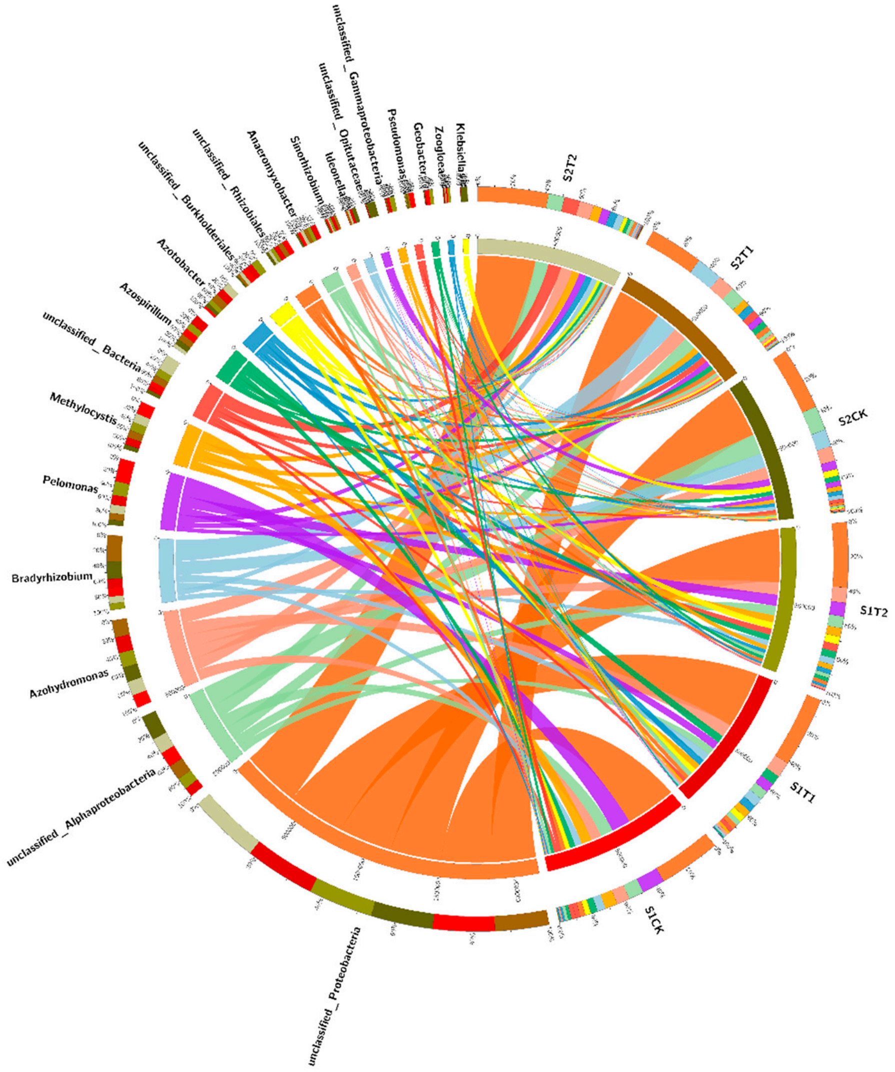

Figure S3 Circos diagram represents the bacterial composition of top genus level under different ventilation conditions.

Table S1 Effects of different ventilation treatments on soil physicochemical properties.

Treatment	AN (mg/kg DW soil)	AP (mg/kg)	AK (mg/kg)
S1CK	6.77a	23.16a	25.49a
S1T1	6.77a	22.77a	27.46a
S1T2	6.94a	22.44a	26.13a
S2CK	6.65b	19.60b	24.58c
S2T1	7.70a	21.49a	32.54a
S2T2	6.65b	20.00ab	29.49b

Abbreviations: S1, peanut growing stage of anthesis; S2, peanut growing stage of podding; CK, no aeration; T1, the ventilation volume is 1.2 times as the standard; T2, the ventilation volume is 1.5 times as the standard. AN, available nitrogen; AP, available phosphorus; AK, available potassium. The values with the same letter between treatments or columns are not significant differences tested by Fisher's test at $P < 5\%$.
